# Arsenious Treatment for the Removal of Tumors from the Mouth

**Published:** 1899-05

**Authors:** J. L. Mewborn

**Affiliations:** Memphis, Tenn.


ARTICLE V.

### ARSENIOUS TREATMENT FOR THE REMOVAL OF TUMORS FROM THE MOUTH.

DR. J. L. MEWBORN, MEMPHIS, TENN.

Read before the Tennessee Dental Association, Lookout Mountain, July, 1898.

### CASE NO. 1.

In 1876 Maggie H—— (eight years old) was brought to me by her father for the removal of two baby incisors below. On further examination I found all the upper baby teeth firmly in position, and located on the gum above the right canine and baby molars was a liver-colored tumor the size of a nickel; its surface was convex and as slick as glass, with margin as well defined as the setting of a watch crystal. After a few days the surface began to break up into a warty appearance with a tenacious yellow exudate from its surface. My proposition to remove the growth with arsenic was received with strenuous opposition by the half dozen leading physicians whom I had assembled for consultation. Holding my proposition in abeyance, I agreed to try any other remedy they might suggest. On the supposition that tardy replacement of the baby teeth might be an exciting cause, I agreed to and did extract the right central, lateral, canine, first and second molars —all baby teeth. This removed all restraint, and in a week the tumor had extended down and inward to the center of the roof of the mouth, becoming as large as a hickory nut. Next, the whole surface of the tumor was pierced in every direction with a pointed orange wood stick, being dipped in C. P., nitric acid before each puncture. This seemed to stimulate vascular action, and caused the whole surface to sprout out, giving it the appearance of a cauliflower. After the third day this treatment was abandoned. By this time three weeks had been wasted in delay and treatment, which had only stimulated its growth until now the lips could not cover it nor the teeth be brought together. The child was beginning to show and feel the lack of proper nourishment. The nasty thing was growing at a fearful rate. The father fully realized the exigency of the case when I assured him that heroic treatment could be no longer delayed. The surgeons had insisted that the knife and bond forceps were the last resort. I explained to the father that this meant chloroform, the loss of a great deal of blood, the destruction of bone and germs of teeth sufficient to disfigure the child for life, together with fever and possibly erysipelas for several weeks. "Now, shall we adopt this or my remedy, which is arsenic—a virulent poison?" To my gratification, he replied: "Doctor, I have watched you closely. I believe you know what you are about. If you say put arsenic in there, why slap it in, and I'll back you." In less than a minute the whole operation was done. After piercing the lobulated structure of the tumor to its center, I carried to this point on a forked instrument a small pellet of cotton saturated with twice the amount of arsenious paste used for destroying the nerve in a tooth, nothing more. In five days the whole thing sloughed away, leaving a clean hole down to the exposed crowns of the advancing permanent teeth. In three weeks this was filled with new tissue. No pain was inflicted, not a drop of blood lost, no reactionary fever supervened, and the child only stopped playing long enough to spit the thing out. Ten years afterwards, when she was eighteen years old, I made a cast of her mouth, which shows a perfect arch and complete denture.

### CASE NO. 2.

A few years after this a negro woman reported that while having a lower tooth extracted the forceps suddenly flew up, striking the right upper central with great force. Soon afterwards a tumor began to grow from the injured pericementum, which continued to grow for a year. Several teeth had been removed to make room for it. When it came into my hands it had protruded from the alveolus directly to the front, and looked like an Irish potato sticking out between the lips. This was treated in similar manner, with good results.

### CASE NO. 3.

The third case was that of an old market woman who reported with a firm knotty growth from the periosteum of the lower canine tooth. This projected against the lower lip, and was the size of a filbert. One application of arsenic, as in No. 1, made a permanent cure.

### CASE NO. 4.

On the 11th day of May, last, Mrs. H——, of Ripley, Miss., came to me with a lobulated tumor, the size of one half of a walnut, growing from the pericementum of the upper right canine, which had been removed, hoping to arrest the growth. It was predunculated, as most of these tumors appear after taking on rapid growth. It was the 11th of May that I made the arsenical application, just as in Case No. 1. On the 14th I removed the blackened mass by manipulating it with a pair of foil pliers, bringing with it one or two long filaments, resembling roots, which penetrated deep into the still empty tooth socket. This patient returned home on the 16th, and, like the others, had no pain, no looss of blood, no destruction of healthy tissues, nor any subsequent effect which would give the least annoyance whatever.

In making this brief report of these four cases, I have purposely avoided all characteristic indications which would aid in any way to determine whether they should be classified as vascular, fleshy, glandular, fibrous, cartilaginous, osseous, or cystic tumors. These indications are not reliable, because they are not uniform in the different stages of development or growth of the tumor; therefore, they do not in every case indicate or determine the course of treatment. As the most reliable guide for the adoption of the arsenical treatment, I would lay down the following rule: On any growth or tumor, either sessile or pedunculated, which arises from the alveolar region of the upper or lower jaw—which has a distinct line of demarcation, and can be punctured to its center with a spear-pointed instrument—use the arsenic without hesitation. If there is no line of demarcation, but a general infiltrated appearance of all the surrounding parts, send the patient to the surgeon with your compliments.

If the tumor cannot be penetrated with an exploring needle, it is cystic or osseous, and would require the gouge and burring engine to break it down. But, Mr. President, I am billed for a report of cases, and not a paper on tumors.—*The Dental Headlight.*

